# Surgical Cases in Mercy Hospital

**Published:** 1872-08-01

**Authors:** 


### SURGICAL CASES IN MERCY HOSPITAL.

### Service of Prof. Andrews.

#### STONE IN THE BLADDER.

J—— C——, aged nine years, has been affected for two years with inflammation of the bladder. He was admitted in a state of exhaustion, and with the penis partly covered with large pustules. After a few days' tonic treatment and rest, a sound was introduced and a calculus discovered. The second day after he was etherized and the stone removed by the lateral operation. It proved to be a fine mulberry calculus (oxalate of lime) about one and a quarter inches in diameter. At the tenth day after the operation the boy is walking about, and seems out of danger.

#### EXSECTION OF THE SHOULDER.

B—— B——, a colored sailor, entered with a chronic ulceration, and fistula on the right shoulder. From the general appearance it was believed that the upper portion of the shaft of the humerus was carious, yet the length and tortuousness of the fistulous tracks was such, that it was impossible to obtain positive proof by the probe. The shoulder joint had a certain amount of motion which was painless and free from any trace of crepitus even upon pressure. From this fact it was hoped that the joint itself was not involved.

The patient was etherized and an exploratory incision made for the introduction of the finger. The digital touch showed that the whole upper extremity of the humerus, joint included, was carious. The incisions were therefore extended freely and three and a half inches of the bone excised. In doing this, care was taken to preserve the periosteum, so as to favor the reproduction of the bone. The articular surface of the scapula also partook of the caries, and was freely trimmed with the gouge. The wound was treated with antiseptic dressings, and did well. It is now about five weeks since the operation, and the patient is able to walk about, and amuses himself by sweeping his own room.

CARIOUS ANKLE HEALED WITHOUT OPERATION.

W—— R——, a laborer, received a crushing injury from the fall of a quantity of brick, which produced a bad comminuted fracture of the lower part of the tibia and fibula. From the ankle joint three or four inches upward the bones were reduced to numerous small pieces like a bag of broken stones. Adjustment was very difficult, on account of the impaction of some of the sharp and displaced fragments, but was accomplished at last under the influence of ether. Cold water and ice bags were freely used to combat the inflammation, and after a few weeks the patient left the hospital with a union, but the bone weak and inflamed. About three weeks after he was brought in again with a foul discharging abscess of the ankle joint, a severe erysipelas of the foot and leg, and great prostration, accompanied with a muttering delirium, and symptoms of septicmæia. The probe showed that the whole ankle joint was in a condition of caries.

As he was so weak that I believed the shock of an amputation would be fatal, I determined to see if he could not be improved in his condition before operating. For this purpose he was given tincture of iron and quinine freely. The erysipelatous surface was washed every three hours with the following lotion:

℞ Zinci chloridi, ʒ j.

Acid carb. cryst. ʒ ij.

Aquæ, f ℥ x.

Misce.

For the joint, full antiseptic treatment was resorted to, that is to say, all the fistulas were thoroughly injected twice a day with a solution of carbolic acid, ten grains to the ounce, and the orifices were sealed by laying on them cotton compresses dipped in

℞ Ol. ricini, f ℥ x.

Acid carbol. cryst. ℥ j.

Misce.

The patient responded finely after a few days of anxious doubt. The suppuration was reduced to a small serous discharge, the erysipelas subsided, and the patient turned back from the edge of the grave. Finding that the vigor was steadily on the increase, I postponed the amputation from week to week, so as to have him in as good a condition as possible, keeping up all the treatment. At the end of about ten weeks the fistulas were completely healed, and all signs of caries obliterated. The joint of course was anchylosed, and the patient was cured without operation.

I previously cured a carious elbow in the same way, but the progress was slower, occupying a portion of ten months. It should not be imagined, however, that this attempt would always be successful. The shoulder joint, whose excision is mentioned above, was treated for some time in the same way, with the effect of reducing the suppuration, but accomplishing no healing, and the operation became a necessity, but the splendid success of the plan in the two cases of the elbow and the ankle show that results of the utmost importance can sometimes be obtained by full antiseptic treatment.

SENILE GANGRENE, AMPUTATION, DEATH.

Mrs. ——, aged eighty years, lost the lower half of the right leg some time ago, by senile gangrene, for which the member was amputated. She was now admitted with the same trouble in the remaining leg. Her extreme age and exhaustion seemed to render amputation hopeless. She was therefore placed on the use of tonics, etc., and the mortified limb was infiltrated with a ninety-five per cent. solution of carbolic acid, introduced at numerous points by means of a hypodermic syringe, to prevent decomposition. Under this treatment she improved so much that I ventured to amputate five inches below the knee. She proved too weak, however, and died the fourth day, of exhaustion.

CONCEALED VARIX.

Mary H—— was admitted for a moderate sized ulcer upon the front of the leg. After treatment in vain several months by every possible method, including skin grafting, I determined to cover it by flaps brought in from either side. The patient was etherized, the ulcer dissected out, and the flaps brought in. The operation revealed the fact that there was in the deeper parts of the fatty tissue a perfect network of enlarged veins, which gave no signs of their existence to external inspection. These vessels had walls thicker than ordinary arteries, and were so numerous that the cutting of two flaps each an inch and a quarter long in the skin and superficial fascia proved an exceedingly bloody operation. There has not been time enough yet to see the result, but it is an extremely interesting question, whether certain chronic ulcers are due to concealed varices beneath their floors.

